# Metastatic renal cell carcinoma presenting as jaundice with biliary and gastric outlet obstruction. A case report

**DOI:** 10.1093/jscr/rjaa591

**Published:** 2021-01-25

**Authors:** Ramiz Iqbal, Elvina Wiadji

**Affiliations:** Department of Surgery, John Hunter Hospital, Newcastle, New South Wales, Australia; Department of Surgery, John Hunter Hospital, Newcastle, New South Wales, Australia

## Abstract

Renal cell carcinoma (RCC) can be an aggressive malignancy that has a propensity to spread to atypical locations, most commonly to lung, bone, lymph node. RCC presenting as obstructive jaundice with gastric outlet obstruction has rarely been cited in literature. This study presents a case of advanced RCC in a patient with obstructive jaundice and associated gastric outlet obstruction from a large right renal RCC with malignant retrocaval lymphadenopathy invading the duodenum and distal common bile duct. The patient underwent anterograde stenting of the biliary system via a percutaneous transhepatic cholangiography and an insertion of a duodenal stent. Immunotherapy was commenced and the patient was discharged home. This case highlights the importance of a multi-disciplinary team approach to the management of a complex surgical patient.

## INTRODUCTION

Renal cell carcinoma (RCC) can be an aggressive malignancy that has a propensity to spread to atypical locations, most commonly to lung, bone and lymph node [1]. RCC presenting as obstructive jaundice has been reported infrequently from metachronous pancreatic or ampullary metastasis. Though nodal metastasis is not uncommon, the presenting features of jaundice and gastric outlet obstruction are rare. This case study aims to highlight this rare presentation of RCC and demonstrates the importance of a multidisciplinary approach to its management.

## Case

A 56-year-old male, smoker, presents to our emergency department with an insidious onset of malaise, early satiety, weight loss, jaundice and a 2-day history of epigastric pain. He was observed to be jaundiced and cachectic with a palpable right upper quadrant mass on abdominal examination.

**Figure 1:**
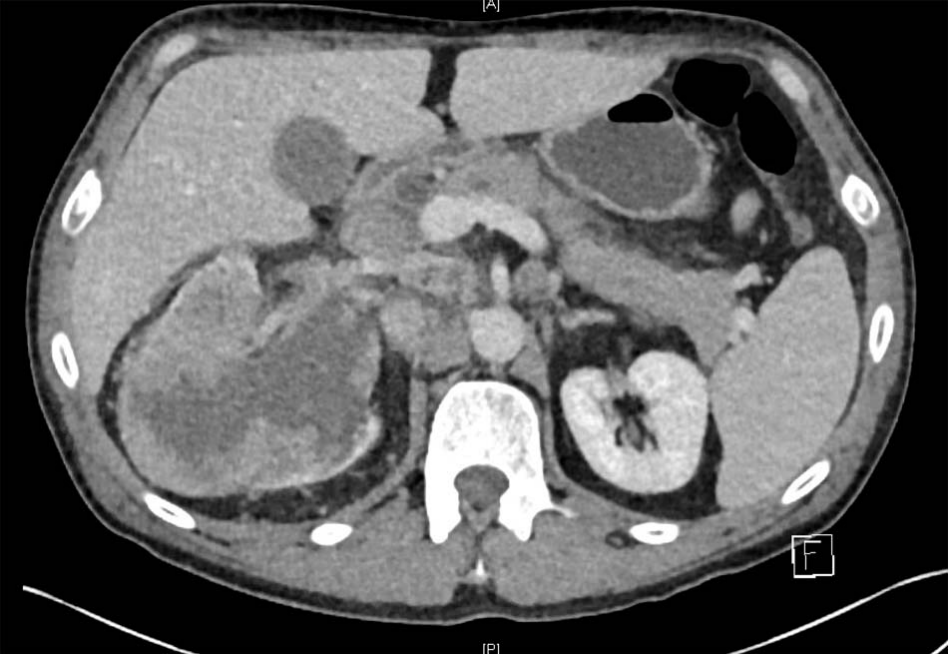
Right renal mass and adrenal/nodal metastasis.

**Figure 2:**
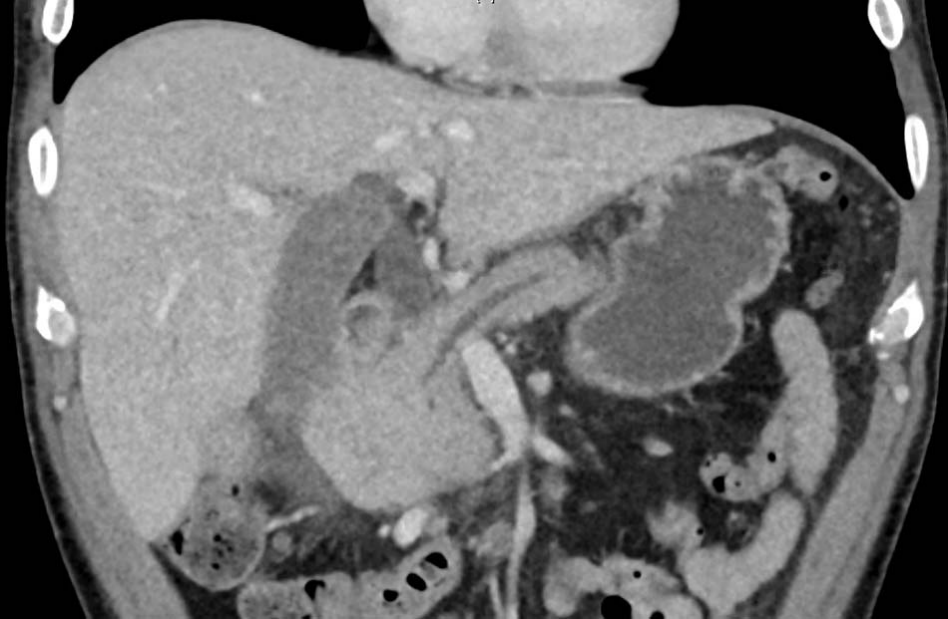
Pancreatic duct dilatation from malignant invasion.

Biochemistry results revealed a lipase and bilirubin of 1105 units/L and 93 umol/L, respectively. Subsequently, a 12.5 × 11.3 cm heterogenous mass arising from the right kidney was found on a contrast enhanced computed tomography (CT) scan (Fig. 1). This mass was associated with a non-contiguous adrenal metastasis ipsilaterally and retrocaval lymphadenopathy adjacent to the pancreatic head resulting in biliary and pancreatic duct dilatation (Fig. 2). A staging CT chest with intravenous contrast was then performed revealing mediastinal lymphadenopathy suggestive of metastasis.

Due to the acuity of the patient’s presentation with pancreatitis along with the need to investigate the underlying aetiology of his presenting symptoms, he was subsequently admitted into the hospital. A multidisciplinary discussion involving general surgery, urology, medical oncology and gastroenterology was conducted to guide patient’s care. Differential diagnosis included RCC, lymphoma and pancreatic cancer. Decision was made to, then, perform an MRCP (Magnetic resonance cholangiopancreatography) revealing a positive double duct sign secondary to malignant invasion of retroperitoneal mass. Furthermore, the second and third part of the duodenum showed reactive changes and luminal distortion indicating gastric outlet obstruction.

**Figure 3:**
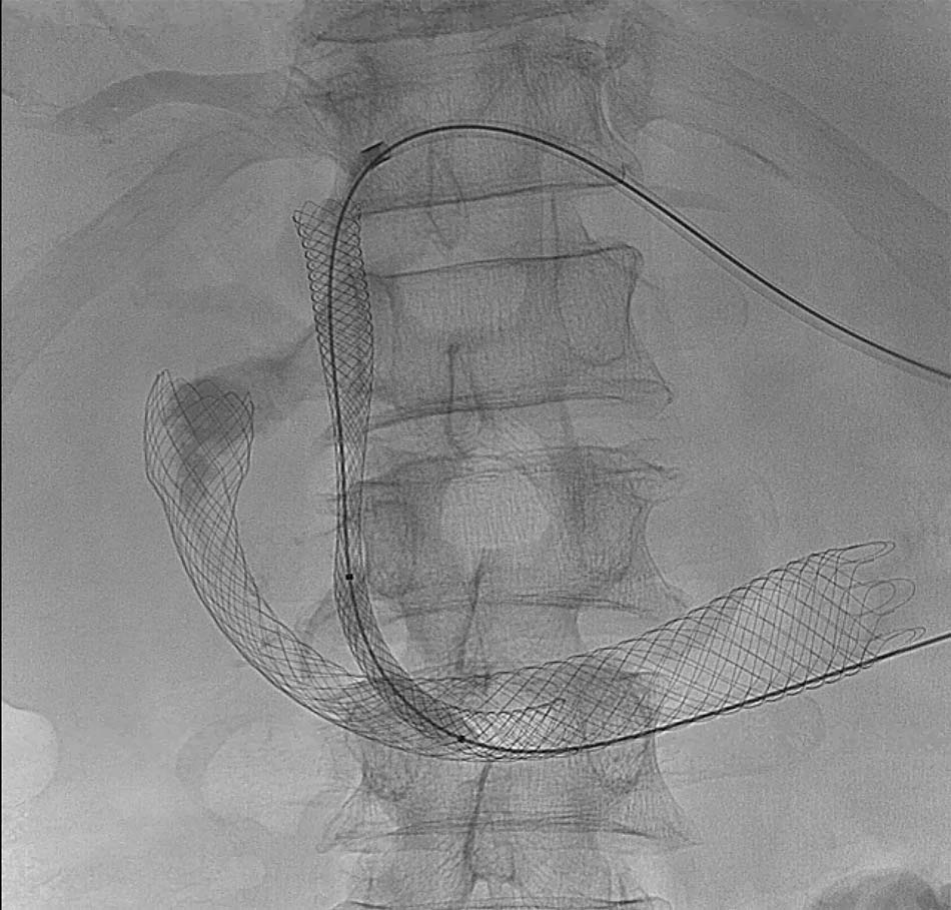
(PTC) Anterograde biliary stent through duodenal wall flex stent.

A retroperitoneal percutaneous biopsy of the retrocaval lymph node mass was performed without any complications. Awaiting histological diagnosis, his gastric outlet obstruction worsened, and hyperbilirubinemia increased to 317 umol/L, developed acute renal failure with creatinine rising to 484 umol/L and progressive anuria. The renal failure was believed to be multifactorial from malignancy and cholemic nephropathy that can be seen in patients with obstructive jaundice [2]. The patient was transferred to HDU (High Dependency Unit) for haemodialysis. Histopathology results confirmed a poorly differentiated high-grade RCC of clear cell subtype. Endoscopic examination performed confirmed an infiltrating obstructive duodenal mass and a self-expanding ‘Wallflex’ duodenal stent was inserted. As retrograde access for biliary stenting was near impossible, a percutaneous transhepatic cholangiography (PTC) was performed with anterograde stenting of the bile duct resolving the hyperbilirubinemia (Fig. 3). Over the following days the patient’s hyperbilirubinemia and renal failure normalized. He recovered uneventfully and was subsequently commenced on immunotherapy prior to discharge.

## DISCUSSION

Thirty percent of RCC are metastatic on presentation. Presently, with the advent of immunotherapies, the mean survival of metastatic disease has increased from 1 to 3 years [1]. RCC is known to metastasis to the pancreas with a number of case reports describing an ampullary or intraductal metastasis resulting biliary obstruction [3–5]. However, to the best of our knowledge, there has been only one other study that describes metastatic RCC causing biliary and gastric outlet obstruction [6].

The management of such lesion posed a diagnostic challenge. Lymphomatous mass resulting in a similar presentation may be responsive to chemoradiotherapy and may be managed differently to a carcinomatous lesion [7]. Therefore, in these cases urgent biopsy is required to guide treatment.

Prioritization of intervention needs to be based on the patient clinical status. Acute clinical deterioration can occur quickly, prompting acute surgical intervention.

Radical surgical intervention in the form of a cytoreductive nephrectomy and pancreaticoduodenectomy in the setting of wide-spread metastasis was collectively agreed to be of limited benefit for this particular patient. A less aggressive approach in the setting of advance disease is also supported by evidence presented in a recent study, the ‘Carmena’ trial which shows no survival benefit of a cytoreductive nephrectomy versus immunotherapy alone [8].

In conclusion, multi-organ failure can result from advanced RCC with biliary and gastric outlet obstruction, a coordinated effort from a multi-disciplinary team is required to direct the acute management of the patient so that a targeted oncological treatment plan can be commenced in a timely manner.

## CONFLICT OF INTEREST STATEMENT

None declared.

## FUNDING

None.
